# Normal weight obesity and the risk of diabetes in Chinese people: a 9-year population-based cohort study

**DOI:** 10.1038/s41598-021-85573-z

**Published:** 2021-03-17

**Authors:** Shaoyong Xu, Jie Ming, Aihua Jia, Xinwen Yu, Jing Cai, Ce Jing, Chun Liu, Qiuhe Ji

**Affiliations:** 1 Department of Health Statistics, Shaanxi Key Laboratory of Free Radical Biology and Medicine and the Ministry of Education Key Lab of Hazard Assessment and Control in Special Operational Environment, School of Public Health, Air Force Medical University, Xi’an, China; 2 Department of Endocrinology, Xiangyang Central Hospital, Affiliated Hospital of Hubei University of Arts and Science, Xiangyang, China; 3 Department of Endocrinology, Endocrinology Research Center, Xijing Hospital, Air Force Medical University, Xi’an, 710032 China; 4 Department of Endocrinology, No.1 Hospital of Yulin, Yulin, China; 5 College of Basic Medicine, Shaanxi University of Chinese Medicine, Xianyang, China

**Keywords:** Diseases, Endocrinology

## Abstract

We evaluated the risk of developing diabetes in Chinese individuals with normal weight obesity (NWO). This 9-year population-based cohort study was based on the China National Diabetes and Metabolic Disorders Survey. A total of 1128 subjects without diabetes were included. Body fat percentage (BF%) was assessed by electrical bioimpedance. NWO was defined as subjects with a normal BMI (< 24 kg/m^2^) and an excess BF% (≥ 24% in men; ≥ 33% in women). Of 1128 individuals, 528 individuals were normal weight non-obese (NWNO), 118 (10.5%) were normal weight obese (NWO), 63 were overweight non-obese (OWNO), and 419 were overweight obese (OWO). During a follow-up of 9.0 years (interquartile range: 8.9–9.3), 113 (10.0%) individuals developed diabetes. The incidence rates of diabetes in NWNO, NWO, OWNO and OWO people were 5.69 (27 cases), 11.30 (12 cases), 3.53 (2 cases) and 19.09 (72 cases) per 1000 person-years, respectively. Cox regression analyses indicated multivariate-adjusted hazard ratios of diabetes in NWO, OWNO and OWO people were 2.110 (95% CI 1.026–4.337, *p* = 0.025), 0.441 (95% CI 0.101–1.928, *p* = 0.232) and 3.465 (95% CI 2.163–5.551, *p* < 0.001), respectively, relative to NWNO people. Chinese people with NWO are at increased risk of developing diabetes. We strongly suggest the incorporation of BF% measurement into the regular physical examination in Chinese medical practice.

## Introduction

During the past three decades, obesity has become a worldwide epidemic that threatens public health. The prevalence of obese and overweight adults increased by 27.5% from 1980 to 2013, going from 857 million in 1980 to 2.1 billion in 2013^1^. Obesity confers a substantial increased risk for morbidity and mortality, particularly from diabetes and cardiovascular diseases^2–5^. However, the current concept of obesity, defined by weight and height measures, has been challenged by evidence indicating this may not accurately identify all obesity-related diseases. This has led to the introduction of a new phenotype, called “normal weight obesity” (NWO) that describes individuals with a normal body mass index (BMI) but increased body fat percentage (BF%)^6^.

Strong evidence exists linking NWO and cardiometabolic dysregulation, cardiovascular disease and overall mortality rates^7^. NWO is associated with insulin resistance, low insulin sensitivity, and high insulin secretion^8^. However, due to a lack of BF% measurements in most large epidemiologic surveys, few cross-sectional studies regarding the association between NWO and diabetes have been conducted and results of these few are controversial^8,9^. For example, one cross-sectional study showed that NWO women had a higher prevalence of fasting hyperglycemia (odds ratio [OR] = 1.63, 95% confidence interval [CI] 1.10–2.42) than lean women^8^, while another study showed that NWO was not significantly associated with high blood glucose (OR: 1.60, 95% CI 0.54–4.76, *p* = 0.395)^9^. More importantly, fewer cohort studies exist^10^. One community-based cohort study reported that subjects with normal BMI but increased BF% had a higher risk of developing diabetes^10^. Thus, population-based cohort studies with long-term follow-up periods are especially needed to clarify the association between NWO and diabetes, and to provide data applicable to the broader population.

We thus conducted a prospective population-based cohort study to evaluate the risk of developing diabetes for a Chinese population with NWO.

## Results

**Figure 1:**
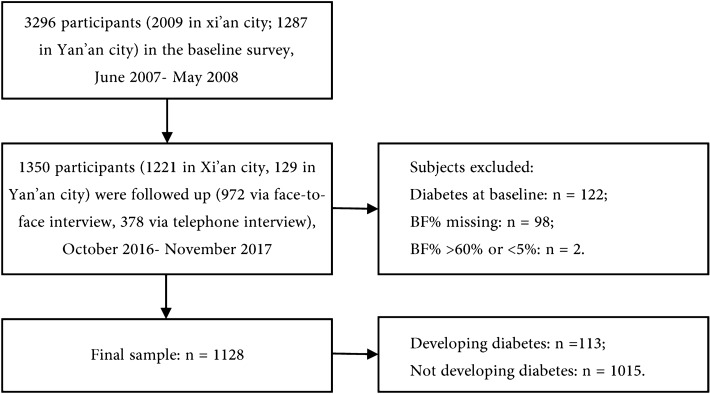
Flowchart of the study population. *BF%* body fat percentage.

The study sample included 1128 individuals without diabetes, with 42.3% male, a mean age of 41.66 years, a mean BMI of 23.65 kg/m^2^, and a mean BF% of 29.17%. During the 9-year (interquartile range: 8.9–9.3) follow-up, 113 (10.0%) individuals developed diabetes, of whom 92 were diagnosed based on the oral glucose tolerance test (OGTT) and 21 diagnoses were based on self-reported diabetes history or use of hypoglycemic drugs (Fig. 1).

Within the study sample, 528 individuals were considered normal weight non-obese (NWNO), 118 (10.5%) were classified as normal weight obese (NWO), 63 were classified as overweight non-obese (OWNO), and 419 were diagnosed as overweight obese (OWO). Compared with NWNO individuals, NWO individuals were more likely to be older, male, to have higher blood pressure, and higher levels of glucose, total cholesterol, LDL cholesterol and triglycerides. Baseline measures are provided according to BMI and obesity phenotypes in Table 1. Compared with individuals who did not develop diabetes, individuals who developed diabetes during follow-up had a higher baseline age, BMI, BF%, blood pressure, glucose level and triglyceride levels (Table 2).

**Table 1:** Baseline characteristics of study participants by obesity phenotypes.

	Total	Normal weight non-obesity	Normal weight obesity	Overweight non-obesity	Overweight obesity	p value
*N*	1128	528	118	63	419	
**Demographic characteristics**						
Age, years	43.89 ± 12.35	41.66 ± 12.71	42.86 ± 12.12	48.83 ± 11.21	46.26 ± 11.48	< 0.001
Male, n (%)	477/1128 (42.3)	171/528 (32.4)	56/118 (47.5)	43/63 (68.3)	207/419 (49.4)	< 0.001
**Educational level, n (%)**						
College or above	343/1120 (30.6)	203/525 (38.7)	29/117 (24.8)	20/62 (32.3)	91/416 (21.9)	< 0.001
Smoking history, n (%)	263/1127 (23.3)	101/528 (19.1)	23/117 (19.7)	26/63 (41.3)	113/419 (27.0)	< 0.001
Drinking history, n (%)	254/1120 (22.7)	90/525 (17.1)	31/117 (26.5)	23/62 (37.1)	110/416 (26.4)	< 0.001
Physical activity, n (%)	450/1121 (40.1)	223/527 (42.3)	39/117 (33.3)	27/62 (43.5)	161/415 (38.8)	0.272
Family history of diabetes, n (%)	133/1128 (11.8)	66/528 (12.5)	12/118 (10.2)	13/63 (20.6)	42/419 (10.0)	0.088
Body weight, kg	61.89 ± 10.82	54.88 ± 6.94	59.42 ± 6.76	68.61 ± 7.44	70.38 ± 9.54	< 0.001
Body mass index, kg/m^2^	23.65 ± 3.29	21.09 ± 1.74	22.75 ± 1.23	25.25 ± 1.04	26.88 ± 2.34	< 0.001
Body fat percent, %	29.17 ± 7.81	24.36 ± 5.17	31.43 ± 4.97	24.65 ± 5.12	35.27 ± 7.00	< 0.001
**Clinical characteristics**						
Systolic blood pressure, mm Hg	119.42 ± 19.66	113.18 ± 17.97	115.99 ± 16.63	123.63 ± 15.13	127.65 ± 20.01	< 0.001
Diastolic blood pressure, mm Hg	75.24 ± 11.23	71.82 ± 9.91	74.50 ± 11.18	75.67 ± 9.63	79.72 ± 11.51	< 0.001
Fasting plasma glucose, mmol/L	5.10 ± 0.65	5.03 ± 0.65	5.06 ± 0.64	5.09 ± 0.66	5.21 ± 0.66	< 0.001
2 h postprandial glucose, mmol/L	6.02 ± 1.55	5.76 ± 1.47	5.76 ± 1.34	5.89 ± 1.39	6.42 ± 1.64	< 0.001
Total cholesterol, mmol/L	4.71 ± 0.91	4.51 ± 0.88	4.71 ± 0.88	4.74 ± 0.86	4.95 ± 0.92	< 0.001
LDL cholesterol, mmol/L	2.64 ± 0.75	2.47 ± 0.73	2.69 ± 0.67	2.66 ± 0.65	2.85 ± 0.77	< 0.001
Triglyceride, mmol/L	1.53 ± 1.09	1.21 ± 0.82	1.37 ± 0.78	1.79 ± 1.09	1.95 ± 1.30	< 0.001

*LDL* low-density lipoprotein.

**Table 2:** Baseline characteristics of study participants by development of diabetes.

Variable	Developing diabetes	Not developing diabetes	*p* value
*n*	113	1013	
**Demographic characteristics**			
Age, y	48.92 ± 10.43	43.31 ± 12.41	< 0.001
Male, n (%)	54/113 (47.8)	423/1014 (41.7)	0.229
**Educational level, n (%)**			
College or above	26/112 (23.2)	317/1007 (31.5)	0.229
Smoking history, n (%)	29/113 (25.7)	234/1013 (23.1)	0.558
Drinking history, n (%)	25/112 (22.3)	229/1007 (22.7)	1.000
Physical activity, n (%)	49/113 (43.4)	401/1007 (39.8)	0.480
Family history of diabetes, n (%)	20/113 (17.7)	113/1014 (11.1)	0.046
Body weight, kg	67.25 ± 11.37	61.31 ± 10.57	< 0.001
Body mass index, kg/m^2^	25.61 ± 3.27	23.44 ± 3.96	< 0.001
Body fat percent, %	33.13 ± 8.17	28.74 ± 7.65	< 0.001
**Clinical characteristics**			< 0.001
Systolic blood pressure, mm Hg	126.95 ± 17.87	118.56 ± 19.67	< 0.001
Diastolic blood pressure, mm Hg	79.91 ± 10.25	74.71 ± 11.21	< 0.001
Fasting plasma glucose, mmol/L	5.23 ± 0.69	5.09 ± 0.65	0.027
2 h postprandial glucose, mmol/L	7.03 ± 1.67	5.90 ± 1.49	< 0.001
Total cholesterol, mmol/L	4.81 ± 0.82	4.70 ± 0.92	0.218
LDL cholesterol, mmol/L	2.73 ± 0.80	2.64 ± 0.75	0.187
Triglyceride, mmol/L	2.10 ± 1.58	1.47 ± 1.00	< 0.001

*LDL* low-density lipoprotein.

The incidence rates of diabetes in NWNO, NWO, OWNO and OWO people were 5.69 (27 cases), 11.30 (12 cases), 3.53 (2 cases) and 19.09 (72 cases) per 1000 person-years, respectively. Cox regression analyses indicated multivariate-adjusted hazard ratios (HRs) of diabetes in NWO, OWNO and OWO people were 2.110 (95% CI 1.026–4.337, *p* = 0.025), 0.441 (95% CI: 0.101–1.928, *p* = 0.232) and 3.465 (95% CI 2.163–5.551, *p* < 0.001), respectively, compared to NWNO people. In male participants, NWO was not significantly associated with diabetes compared to NWNO (HR: 1.163, 95% CI 0.314–4.301, *p* = 0.821), while in female participants, a significant association between NWO and diabetes occurred (HR: 3.102, 95% CI 1.372–7.014, *p* = 0.007) (Table 3).

**Table 3:** Cox regression analysis of obesity phenotypes in predicting type 2 diabetes mellitus in total, male, and female participants.

	Normal weight non-obesity	Normal weight obesity	Overweight non-obesity	Overweight obesity
**Total participants**				
Cases/participants, n/N	27/527	12/118	2/63	72/419
Incidence, %	5.1	10.2	3.2	17.2
Crude HR (95% CI)	1.000	2.087 (1.052–4.143)	0.505 (0.120–2.129)	4.011 (2.553–6.303)
*p* value		0.035	0.352	< 0.001
Adjusted HR (95% CI)	1.000	2.110 (1.026–4.337)	0.441 (0.101–1.928)	3.465 (2.163–5.551)
*p* value		0.025	0.232	< 0.001
**Male participants**				
Cases/participants, n/N	10/171	3/56	1/43	40/207
Incidence, %	5.8	5.4	2.3	19.3
Crude HR (95% CI)	1.000	1.086 (0.294–4.016)	0.332 (0.042–2.635)	4.063 (1.970–8.382)
*p* value		0.902	0.297	< 0.001
Adjusted HR (95% CI)	1.000	1.163 (0.314–4.301)	0.307 (0.039–2.427)	4.167 (2.014–8.620)
*p* value		0.821	0.263	< 0.001
**Female participants**				
Cases/participants, n/N	17/356	9/62	1/20	32/212
Incidence, %	4.8	14.5	5.0	15.1
Crude HR (95% CI)	1.000	2.906 (1.288–6.556)	0.931 (0.124–7.016)	3.824 (2.105–6.947)
*p* value		0.010	0.945	< 0.001
Adjusted HR (95% CI)	1.000	3.102 (1.372–7.014)	0.779 (0.103–5.897)	3.403 (1.847–6.269)
*p* value		0.007	0.809	< 0.001

*CI* confidence interval.

*Hazard ratio and 95% CI were calculated using a backward stepwise method. The covariables were age, (gender), cigarette smoking, alcohol drinking, physical activities, family history of diabetes and baseline blood glucose levels.

Sensitivity analyses were conducted by excluding participants in the study site with a low follow-up rate or by excluding participants with baseline pre-diabetes (n = 218); results were similar to the full data set in both cases. Excluding participants with baseline metabolic syndrome (MS) (n = 299) or participants with only a telephone interview (n = 340), gave similar HRs but effects were no longer statistically significant, partly due to sample size insufficiency. Details are given in Table 4.

**Table 4:** Sensitivity analyses.

	Normal weight non-obesity	Normal weight obesity	Overweight non-obesity	Overweight obesity
Population in sites with high follow-up rates*	**(n** = **1044)**			
n/N, %	25/490 (5.1)	12/111 (10.8)	2/59 (3.4)	70/384 (18.2)
Crude HR (95% CI)	1.000	2.177 (1.092–4.341)	0.521 (0.123–2.200)	4.025 (2.538–6.383)
Adjusted HR (95% CI)	1.000	2.280 (1.143–4.548)	0.422 (0.100–1.791)	3.756 (2.362–5.973)
**Population without baseline pre-diabetes**	**(n** = **910)**			
n/N, %	15/447 (3.4)	9/96 (9.4)	2/52 (3.8)	43/315 (13.7)
Crude HR (95% CI)	1.000	2.024 (1.306–7.002)	0.948 (0.215–4.176)	4.982 (2.707–9.170)
Adjusted HR (95% CI)	1.000	3.113 (1.344–7.212)	0.756 (0.171–3.351)	4.750 (2.571–8.778)
**Population without baseline MS**	**(n** = **829)**			
n/N, %	23/479 (4.8)	9/98 (9.2)	2/46 (4.3)	27 /206 (13.1)
Crude HR (95% CI)	1.000	2.012 (0.902–4.653)	0.876 (0.343–3.872)	2.769 (1.582–5.220)
Adjusted HR (95% CI)	1.000	1.845 (0.883–4.467)	0.665 (0.143–3.136)	2.781 (1.524–5.189)
**Population with only office follow-up**	**(n** = **788)**			
n/N, %	23/360 (4.2)	10/84 (11.9)	2/50 (4.0)	57/294 (19.4)
Crude HR (95% CI)	1.000	1.792 (0.855–3.754)	0.460 (0.109–1.946)	3.697 (2.295–5.956)
Adjusted HR (95% CI)	1.000	1.869 (0.890–3.921)	0.402 (0.095–1.708)	3.662 (2.272–5.903)

*RR* relative risk, *CI* confidence interval.

*Hazard ratio and 95% CI were calculated using a backward stepwise method. The covariables were age, gender, cigarette smoking, alcohol drinking, physical activities, family history of diabetes, and baseline blood glucose levels.

## Discussion

We previously reported that the optimal foot-to-foot BIA-measured BF% cutoff in Chinese people is 24% for men and 33% for women. The prevalence of NWO is 7.39% based on these criteria^11,12^. In the present study, we conduct a 9-year longitudinal survey and show that Chinese people with NWO had an approximately two times greater risk of developing diabetes (HR: 2.110, 95% CI 1.026–4.337, *p* = 0.025) compared to NWNO controls. Overweight people with normal BF% did not have an increased risk of diabetes (HR: 0.441, 95% CI 0.101–1.928, *p* = 0.232). The strengths of this study include that it is a population-based sample, has a long-term follow-up period, diagnosed diabetes based on OGTT, excluded individuals with diabetes at baseline, and adjusted for several potentially confounding factors.

Our results showed that people with NWO have a significantly increased risk of developing diabetes. Only a few cross-sectional studies have previously explored the association between NWO and insulin resistance, hyperglycemia or diabetes, but the results were equivocal^8,9^. For example, Madeira et al. studied 1222 males and females aged 23–25 years and showed that NWO was significantly associated with a homeostasis model assessment-insulin resistance (OR = 3.81, 95% CI 1.57–9.82) but not with high blood glucose (OR: 1.60, 95% CI 0.54–4.76, *p* = 0.395)^8^. In contrast, a cross-sectional study conducted by Marques-Vidal et al*.* with 3213 women and 2912 men aged 35–75 years in Switzerland, suggested that NWO women had a higher prevalence of fasting hyperglycemia (OR = 1.63, 95% CI 1.10–2.42) than lean women^9^. Our results from the current study are similar to a previous Chinese cohort study in which Zhao et al. conducted a community-based study of 1857 normal glucose tolerance subjects that were followed for 44.57 months. Compared with individuals having normal BF% and BMI, subjects with normal BMI but increased BF% had a higher risk of developing diabetes (Relative risk [RR]: 4.790, 95% CI 1.061–21.621)^10^. Differences in RRs between Zhao et al. and our study might be due to differences in study design, duration of follow up, control group, and baseline metabolic state of the subjects included.

In addition, we found that overweight people with normal BF% did not have an increased risk of diabetes (HR: 0.441, 95% CI 0.101–1.928, *p* = 0.232). The results agree with those of Zhao et al.^10^, who found that those with stable normal BF% and abnormal BMI did not have a significantly higher risk of developing diabetes (RR = 2.838, 95% CI 0.293–27.537). The possible existence of *healthy obesity* has been a subject of debate for many years because some overweight and obese individuals are found to have a metabolically healthy status^13^. However, meta-analyses indicates that adults with metabolically heathy obesity (MHO) have substantially increased risks of developing diabetes and cardiovascular events compared with metabolically healthy normal-weight adults^14,15^, and prospective evidence does not indicate that healthy obesity is a harmless condition. Thus, data corroborate the concept that MHO is one step in the process of obesity whereby individuals are likely to become metabolically unhealthy^16^. Nonetheless, the key point is that obesity judged by BMI is problematic, because BMI does not take into account a person’s bone, muscle, or fat proportions, and thus fat mass and fat-free mass are not differentiated with BMI^17^. In fact, a person with a higher BMI but normal BF% is more likely to take part in strength training or be an athlete. For example, in the National Football League (NFL), 97% of all players have a BMI of ≥ 25.0 kg/m^2^, but based on BF% only 8.9% of these athletes are obese and 14.5% have below-normal BF%^18^. Thus, BF% is a more accurate measure for identification of obesity-related diabetes risk in populations such as athletes, and clinical focus should be on BF% rather than BMI.

A previous nationwide population-based cohort study with a mean follow-up of 8.8 years showed that NWO was associated with high prevalence of cardiometabolic dysregulation, metabolic syndrome, and cardiovascular risk factors. In women, NWO is independently associated with increased risk for cardiovascular mortality^7^. Those findings, together with our own, drew our attention to NWO, as a special phenotype of obesity. Since obesity is defined as a condition where there is an excess of body fat^19^, as opposed to excess weight (in fact, excess BMI), the current concept of diagnosing obesity using BMI has been challenged. Early in 1981, Ruderman et al. defined a novel phenotype of obesity as metabolically obese normal weight (MONW). They described individuals with MONW as characterized by normal body weight and BMI, but with hyperinsulinemia and possibly an increase in fat cell size^20^. However, not all persons with normal body weight but high BF% have this cluster of metabolic abnormalities, and, in 2005, De Lorenzo et al. formally introduced the term, NWO. We would emphasize that NWO is of greater concern than MONW, a condition that has been studied for some decades. In contrast to subjects with MONW, who present major metabolic changes that result in signs and symptoms as well as subsequent diagnosis^21^, individuals with NWO are likely undiagnosed and thus unaware of the cardiometabolic risks they face. NWO screening using simple methods (e.g., foot-to-foot BIA) is thus strongly suggested in clinical practice.

Several limitations of the study should be addressed. First, although dual energy X-ray absorptiometry is considered the gold standard for BF% measurement^22^, we used bioelectrical impedance in our study based on its acceptable accuracy, simplicity, lack of radiation, and relatively low cost^23^. Nevertheless, bioelectrical impedance may underestimate upper-body obesity^24^, we had a relatively low follow-up rate (41.0%) for the entire sample. However, after excluding 177 individuals in the low-rate sites, the follow-up rate was 72.6% with a sample size of 1173, which is sufficient for a high-quality cohort study. We also conducted sensitivity analysis by excluding sites with low follow-up rate and showing results were similar. Third, misclassification may have happened in our study, since people could have had changes in their body composition during the follow-up period. This concern is true for most longitudinal studies which use baseline information on the exposure variable. Fourth, unmeasured and residual confounding effects cannot be eliminated although a range of potentially confounding factors were included. For example, information on dietary intake was not collected. Fifth, cases of incident diabetes and overweight non-obesity were small, so relevant conclusions need to be confirmed by future studies with larger sample sizes. Last, our findings from a single-province survey might not be generalizable to the whole country.

In conclusion, we demonstrate that Chinese people with NWO are at increased risk of developing diabetes. Our study shows that BF% constitutes a better measure of obesity assessment than BMI. We strongly suggest incorporation of BF% measurement into the regular physical examination, and NWO screening using simple methods in clinical practice in the Chinese population.

## Methods

### Study sample

This cohort study with a follow-up of 9 years was based on the China National Diabetes and Metabolic Disorders Study (CNDMDS). CNDMDS was a nationwide population-based cross-sectional survey, with aims to investigate the prevalence of diabetes and associated metabolic risk factors in China. The current cohort study included only individuals of CNDMDS in Shaanxi province, Northwestern China. During June 2007 and May 2008, we used a multi-stage stratified sampling method to select a representative sample from Shaanxi province, narrowing down the sample pool in four stages, as detailed previously^25^. Briefly, in the first stage, two cities (Xi’an as capital city and Yan’an as a non-capital city) were selected non-randomly. In the second stage, city districts from cities and rural townships from countryside were randomly selected. In the third stage, street districts from city districts and rural villages from townships were randomly selected. In the last stage, adults ≥ 20 years were selected using stratified sampling methods according to the sex and age distribution.

In total, 3296 subjects (2009 in Xi’an city and 1287 in Yan’an city) were selected as the baseline study sample and were invited to participate in the 9-year follow-up evaluation from October 2016 to November 2017. Of these, 1350 subjects (1221 in Xi’an city and 129 in Yan’an city) agreed and participated in the follow-up evaluation; 972 had a face-to-face evaluation and 378 were evaluated by telephone (21 were deceased). The total follow-up rate was 41.0% in all study sites; after excluding study sites with a low follow-up rate, the rate was 72.6% with a study sample size of 1173. The details of follow-up rate for each study site are described in Table S1. Comparison of baseline data between participants who did and did not undergo follow-up examinations is provided in Table S2. We excluded subjects with diabetes (*n* = 122) diagnosed by oral glucose tolerance test (OGTT) or a history of antidiabetic medications at baseline. We also excluded 98 subjects with missing data for BF% and two subjects with BF% > 60% or < 5%. Ultimately, a total of 1128 subjects were included as our study sample for analysis (Fig. 1).

This study was approved by the Ethics Committee of Xijing Hospital, Affiliated Hospital of Fourth Military Medical University. All participants signed written informed consent prior to data collection. All methods were performed in accordance with the relevant guidelines and regulations.

### Baseline data collection

During 2007–2008, using a standard questionnaire, trained physicians and nurses collected baseline data on demographic characteristics, lifestyle risk factors, personal medical history and family history of diseases^26^. Body size and obesity phenotype were evaluated including body height, body weight, waist circumference and BF% measurement. Body weight was measured in kilograms on a calibrated digital scale and body height was measured using a stadiometer in centimeters with subjects wearing light clothes and without shoes. BF% was assessed by electrical bioimpedance analysis (BIA) using the Tanita TBF-300 body composition analyser (Tanita Corporation, Tokyo, Japan) at baseline during 2007–2008. An OGTT was performed on all subjects. Subjects without history of diabetes were administered a standard 75-g glucose solution after at least 10 h of overnight fasting. For security reasons, subjects with a self-reported history of diabetes were given a steamed bun with approximately 80 g of complex carbohydrates. A hexokinase enzymatic method was used to measure serum glucose. Plasma lipid profiles, including total cholesterol, serum triglyceride and low-density lipoprotein (LDL) cholesterol were also measured from fasting blood samples by the enzymatic method. All laboratory measurements were conducted under conditions of a standardization and certification program^25^.

### Assessment of obesity phenotype

Subjects were stratified into four groups according to baseline BMI and BF%. BMI was calculated by dividing body weight in kilograms by the square of body height in meters. The normal weight obese (NWO) group contained subjects with a normal BMI (< 24 kg/m^2^) and an excess BF% (≥ 24% in men and ≥ 33% in women)^11^. Subjects with a normal BMI (< 24 kg/m^2^) and normal BF% (< 24% in men and < 33% in women) were placed in the normal weight non-obese (NWNO) group as controls. Subjects with a BMI ≥ 24 kg/m^2^ were considered overweight, and included both overweight (24 kg/m^2^ ≤ BMI < 28 kg/m^2^) and obese (BMI ≥ 28 kg/m^2^) subjects. Overweight subjects with a normal BF% were placed in the overweight non-obese (OWNO) group, and overweight subjects with an excess BF% were in the overweight obese (OWO) group.

### Assessment of covariates

Alcohol drinking was defined as consuming at least 30 g of alcohol per week for at least a year. Individuals that had consumed at least 100 cigarettes during their lifetime were classified as cigarette smoking. Regular leisure-time physical activity was defined as participation in moderate to vigorous activity for 30 min or more per day on at least 3 days a week. Education level was categorized as (1) below college or (2) college and above. Family history of disease was defined as at least one of parents or siblings diagnosed with a disease in their lifetime and based on self-reporting^25^. Blood pressure was measured using a mercury sphygmomanometer in a sitting position in the morning after at least 5 min of rest. Two consecutive measurements were performed and the mean of the measurements recorded.

### Follow-up evaluation

The primary outcome was the incidence of diabetes with a follow-up of 9 years. For subjects receiving face-to-face evaluation at the follow-up survey, OGTT was performed to evaluate diabetes status, as described above. According to World Health Organization diagnostic criteria^27^, diabetes is defined as the use of antidiabetic medications, a fasting glucose level ≥ 7.0 mmol/l, or a 2-h glucose level ≥ 11.1 mmol/l after a 75 g OGTT. Pre-diabetes was defined as fasting glucose level between 6.1 and 6.9 mmol/l, and/or a 2-h glucose level between 7.8 and 11.0 mmol/l after a 75 g OGTT. For individuals receiving follow-up interviews by telephone evaluation, diabetes was diagnosed by self-reporting.

### Statistical analysis

Sample size requirement was estimated in advance using PASS 11.0 (NCSS, LLC, Utah, USA). Based on a previous study that indicated NWO individuals had a 4.8-fold increased risk of diabetes (95% CI 1.1–21.6)^10^, a sample size of 106–425 participants was planned to provide an 80% power with 95% confidence intervals (α = 0.05), which corresponds to HRs of 2.0–4.0 (P0 was estimated as 5%).

Data were summarized as the mean ± standard deviation (SD) for normally distributed data, the median with interquartile range for skewed variables, or percentages, as suitable. Differences between groups in continuous variables were analyzed by a t-test or analysis of variance (ANOVA) as appropriate. Differences for enumeration data were assessed by the chi-square test of independence.

The incidence of diabetes was calculated for the NWNO, NWO, OWNO and OWO groups. To identify the impact of NWO on the risk of diabetes, a cox regression analysis was done and hazard ratios (HRs) with corresponding 95% CI were obtained using a backward stepwise method. In addition, sensitivity analyses were conducted to assess the robustness of the results by re-running all the models with a subset of individuals excluded. All statistical analyses were performed using SPSS 18.0 (SPSS Inc., Chicago, IL, USA). All reported *P* values are two-tailed, and statistical significance was set at a *P*-value < 0.05.

### Ethics approval and consent to participate

This study was approved by the Ethics Committee of Xijing Hospital, Affiliated Hospital of Fourth Military Medical University. All participant signed written informed consent prior to data collection.

## Supplementary Information

Supplementary Information
