# Supplementary material for: Normal weight obesity and the risk of diabetes in Chinese people: a 9-year population-based cohort study
Source: Sci Rep. 2021 Mar 17;11:6090. doi: 10.1038/s41598-021-85573-z (PMC7969601; doi:10.1038/s41598-021-85573-z)
Supplement: Supplementary file 1 — Supplementary Information [file 41598_2021_85573_MOESM1_ESM.docx]

**Normal weight obesity and the risk of diabetes in Chinese people: a 9-year population-based cohort study**

Shaoyong Xu^1,2*^, Jie Ming^3*^, Aihua Jia^4*^, Xinwen Yu^3^, Jing Cai^5^, Ce Jing^3^, Chun Liu^3^, and Qiuhe Ji^3^

**Table S1 Follow-up rate in study sites**

| **No.** | **Study site** | **Baseline** |  | **Face-to-face interview** | |  | **Telephone interview** | |  | **Total** | |
| --- | --- | --- | --- | --- | --- | --- | --- | --- | --- | --- | --- |
|  |  | *n* |  | *n* | **rate, %** |  | *n* | **rate, %** |  | *n* | **rate, %** |
| 1 | Affiliated Primary School of Xi’an Jiaotong University, Xi’an | 51 |  | 26 | 50.98 |  | 23 | 45.10% |  | 49 | 96.08 |
| 2 | Dong-han Village, Hu Country, Xi’an | 215 |  | 125 | 58.14 |  | 66 | 30.70 |  | 191 | 88.83 |
| 3 | Jia-jia-tan Village, Xi’an | 295 |  | 179 | 60.68 |  | 72 | 24.41 |  | 251 | 85.08 |
| 4 | Li-zhi Primary School, Xi’an | 54 |  | 36 | 66.67 |  | 9 | 16.67 |  | 45 | 83.33 |
| 5 | Yan-xiao Village, Chang’an Country, Xi’an | 212 |  | 122 | 57.55 |  | 49 | 23.11 |  | 171 | 80.66 |
| 6 | Xi-tie Community, Lin-tong District, Xi’an | 100 |  | 70 | 60.00 |  | 10 | 10.00 |  | 70 | 70.00 |
| 7 | Fu-ping Country, Xi’an | 111 |  | 57 | 51.35 |  | 18 | 16.21 |  | 75 | 67.57 |
| 8 | No. 2 Chang’an Middle School, Xi’an | 172 |  | 89 | 51.74 |  | 25 | 14.53 |  | 114 | 66.28 |
| 9 | Xi-hang Community, Xi’an | 312 |  | 116 | 37.18 |  | 46 | 14.74 |  | 162 | 51.92 |
| 10 | Xijing Hospital, Xi’an | 93 |  | 33 | 35.48 |  | 12 | 12.90 |  | 45 | 48.39 |
| 11 | Qu-bao Village, Hu Country, Xi’an | 106 |  | 0 | 0 |  | 33 | 31.13 |  | 33 | 31.13 |
| 12 | Dian-ji-chang Community, Xin-cheng District, Xi’an | 118 |  | 0 | 0 |  | 13 | 11.02 |  | 13 | 11.02 |
| 13 | Gan-ting Village, Hu Country, Xi’an | 170 |  | 0 | 0 |  | 2 | 1.18 |  | 2 | 1.18 |
| 14 | Yan’an City | 1287 |  | 129 | 10.02 |  | 0 | 0.00 |  | 129 | 10.02 |
|  | **Total sites** | **3296** |  | **972** | **29.49** |  | **378** | **11.47** |  | **1350** | **40.96** |
|  | **Sites excluding No. 11,12,13 and 14** | **1615** |  | **843** | **52.20** |  | **330** | **20.43** |  | **1173** | **72.63** |

**Table S2 Comparison of baseline data between participants who did (group A) and did not (group B) undergo follow-up examinations**

| Variable | Group A | Group B | *P* value |
| --- | --- | --- | --- |
| *n* | 1370 | 1946 |  |
| **Demographic characteristics** |  |  |  |
| Age, year | 44.48 ±12.56 | 42.98 ± 14.43 | 0.002 |
| Gender (male), n (%) | 576 (43.2) | 789 (41.1) | 0.233 |
| Body weight, kg | 62.30 ± 10.97 | 62.65 ± 11.03 | 0.374 |
| Body mass index, kg/m2 | 23.80 ± 3.38 | 23.92 ± 3.51 | 0.348 |
| Educational level, n/N (%) |  |  | 0.689 |
| College or above | 387/1320 (29.3) | 510/1855 (27.5) |  |
| Family history of DM, n/N (%) | 158/1320 (12.0) | 224/1902 (11.8) | 0.676 |
| Cigarette smoking, n/N (%) | 315/1331 (23.7) | 454/1918 (23.7) | 1.000 |
| Alcohol drinking, n/N (%) | 201/1322 (22.8) | 484/1899 (25.5) | 0.080 |
| Physical activities, n/N (%) | 533/1324 (40.3) | 681/1908 (35.7) | 0.009 |
| **Clinical characteristics** |  |  |  |
| Systolic blood pressure, mm Hg | 120.75 ± 20.51 | 121.09 ± 19.86 | 0.640 |
| Diastolic blood pressure, mm Hg | 75.81 ± 11.41 | 77.39 ± 11.50 | <0.001 |
| Fasting blood glucose, mmol/l | 5.36 ± 1.38 | 5.27 ± 1.43 | 0.032 |
| 2h postprandial glucose, mmol/l | 6.73 ± 3.32 | 6.55 ± 3.27 | 0.121 |
| Serum total cholesterol, mmol/l | 4.75 ± 0.94 | 4.66 ± 0.99 | 0.021 |
| Serum triglycerides, mmol/l | 1.58 ± 1.11 | 1.51 ± 1.08 | 0.077 |
| Serum LDL cholesterol, mmol/l | 2.68 ± 0.77 | 2.62 ± 0.82 | 0.076 |
| Body fat, % | 29.41 ± 8.05 | 30.34 ± 10.45 | 0.014 |

DM: diabetes mellitus; LDL: low-density lipoprotein.
